# The provision of toys to pigs can improve the human-animal relationship

**DOI:** 10.1186/s40813-020-00167-x

**Published:** 2020-11-10

**Authors:** Míriam Marcet-Rius, Patrick Pageat, Cécile Bienboire-Frosini, Eva Teruel, Philippe Monneret, Julien Leclercq, Alessandro Cozzi

**Affiliations:** 1 Physiological and Behavioural Mechanisms of Adaptation Department, IRSEA (Research Institute in Semiochemistry and Applied Ethology), Quartier Salignan, 84400 Apt, France; 2 Semiochemicals’ Identification and Analogs’ Design Department, IRSEA (Research Institute in Semiochemistry and Applied Ethology), Quartier Salignan, 84400 Apt, France; 3 Statistical Analysis Service, IRSEA (Research Institute in Semiochemistry and Applied Ethology), Quartier Salignan, 84400 Apt, France; 4 Animal Experimentation Service, IRSEA (Research Institute in Semiochemistry and Applied Ethology), Quartier Salignan, 84400 Apt, France; 5 Research and Education Board, IRSEA (Research Institute in Semiochemistry and Applied Ethology), Quartier Salignan, 84400 Apt, France

**Keywords:** Environmental enrichment, Human-animal relationship, Play behaviour, Positive emotions, Tail movement, Strange person test

## Abstract

It is now widely recognised that a positive human-animal relationship is beneficial not only for farm animals’ welfare but also for productivity and the quality of products. A better understanding of animal emotions is an important goal in disciplines ranging from neuroscience to animal welfare science, but few reliable tools exist for measuring these emotions. In this study, whether the provision of toys to solicit play behaviour in pigs is associated with a change in the human-animal relationship and the emotional state of pigs was investigated. We involved a group of sixteen mini-pigs housed in an experimental setting and the use of a preliminary test called the ‘strange person’ test. After a Control and a Play session (with medium-sized dog toys, balls with ropes), the strange person test was performed. During the test, a person wearing a colourful overall, a hood, a mask, gloves and boots (unknown person with an odd appearance) entered the pen, where 2 mini-pigs were housed, for a 2-min video recording. The strange person test results after the Play and Control sessions were compared. The results showed that the latency to approach the person (duration in seconds) and the duration for which the pig was distant from the strange person (duration in seconds) were significantly lower after the Play session than after the Control session (Degrees of Freedom =30; Statistic of the F test =39.1; *p* < 0.0001 and Degrees of Freedom =15; Statistic of the F test =54.3; p < 0.0001, respectively). The duration of direct contact (duration in seconds) (Degrees of Freedom =15; Statistic of the F test =14.8; *p* = 0.002), the need to separate the pig from the strange person (frequency) (Degrees of Freedom =30; Statistic of the F test =9.3; *p* = 0.005) and the duration of tail movement (duration in seconds) (Degrees of Freedom =15; Statistic of the F test =12.6; *p* = 0.003) were all significantly higher after the Play sessions than after the Control sessions. Overall, the results suggest a change in the human-animal relationship after the Play sessions: the pigs seemed to be less fearful and more inclined to interact with the strange person, showing a more positive emotional state. This preliminary study suggests that the provision of toys, and more precisely, the opportunity to perform object play behaviour, and sometimes, spontaneously, social play behaviour, can improve the human-animal relationship. Additional research to explore this topic thoroughly may yield interesting results because a positive emotional state of the animals and a good human-animal relationship are essential to ensure good quality of life of farm animals.

## Introduction

A human-animal relationship can be defined as the degree of relatedness or distance between animals and humans [12]. It is widely recognised since many years now that a positive human-animal relationship is beneficial not only for the welfare of farm animals but also for productivity and the quality of animal products [58]. On the one hand, a negative human-animal relationship occurs when negative handling is performed by stockpeople or any other person in contact with farm animals, such as a veterinarian. These negative interactions, like aversive interactions or perceived as threatening (e.g. moving abruptly the animals from one pen to another, tagging or weighting brusquely), can lead to many undesirable repercussions, not only for the animals but also for the industry and the consumers. One important repercussion is the fear of humans, which represents one of the factors for depressed growth and reproductive performance in commercial pigs [24]. In addition, pigs who are fearful of humans are generally the most difficult pigs to handle [22]. According to Waiblinger et al. [58], several studies showed that negative handling increases adrenal weight (indicative of chronic stress), impairs growth and reproductive performance and induces a high fear of humans, both in the laboratory [16, 24, 51] and on commercial farms [20, 21, 25]. For example, one study by Hemsworth et al. [28] with 90 commercial pigs examined the relationships between handling prior to slaughter and some measures of meat quality, and the results showed a negative correlation between the negative interactions received and the plasma glucose concentrations post-slaughter and a positive correlation between both post-slaughter plasma lactate concentrations and the light reflectance of the ham (a high reflectance indicates a pH drop during post-mortem glycolysis, which is linked to PSE meat: pale, soft, exudative) and other factors. Other studies have shown that there is a progressive increase in the potential incidence of pale, soft, exudative (PSE) and dark, firm, dry (DFD) meat in the slaughterhouse plants using more stressful handling systems (e.g. [59]). These results suggested that the behaviour of stockpeople prior to slaughter can influence meat quality.

On the other hand, regular pleasant contact with humans, which fosters a positive human-animal relationship, may result in desirable effects to the physiology, behaviour, health and productivity of farm animals [63]. Several studies showed many positive aspects, such as the reduction of stress reactions of cows during rectal palpation/insemination due to previous positive handling as well as positive, gentle interactions from the person during the procedure [57] and the improved possibility to detect oestrus behaviour in fearful sows after gentle handling [43]. In addition, gentle handling and stroking of dairy cows and heifers showed to decrease their fear of humans [5], reduce cortisol levels [19], and lower their heart rate [50] during different procedures [44]. Rault [45] showed that positive human contact led to sustained cerebrospinal fluid oxytocin elevation in pigs for 120 min, which outlasted the 15-min interaction. Additionally, Rault [45] showed that the frequency of positive interactions was correlated with an increase in cerebrospinal fluid oxytocin, providing a neurophysiological basis for a positive human-animal relationship.

Nevertheless, the question of how human contact can have a positive impact on responses to stressors and productivity is not well understood [63]. In addition, the literature on the ability of farm animals to recognise individual people is inconsistent [63]. Some studies suggested that farm animals respond the same way to different people. Hemsworth [23] compared the response of pigs to two different stockpersons who differed markedly in their nature of contact with pigs. The results suggested that pigs are unable to differentiate between different people and that aversive handling by one person makes the animals fearful of all people. In contrast, other studies [32, 54] suggested that pigs are able to recognise individual people. Thus, further research on this topic may be very useful.

The assessment of human-animal valence is essential to obtain more information about the welfare of farm animals. Many different methods and tests exist to assess the valence of the human-animal relationship and the emotional state of animals, as they are generally linked, such as the human approach test in sows [18], the qualitative behaviour assessment in sows and other species [61], the avoidance distance test in cows and other species [60], tests measuring the approach or avoidance of people (e.g., [27]), visual attention in horses [48], the presence of a stationary human test and the presence of a moving human test in sheep [8], and an adaptation of the methods of behavioural assessment in zoo animals [6].

A better understanding of animal emotions is an important goal in disciplines ranging from neuroscience to animal welfare science, but few reliable tools exist for measuring these emotions [4, 10]. Play behaviour is generally recognised as a trigger of positive emotions in mammals [30, 39], and previous studies consistently suggested that a high tail movement duration (a behaviour often seen during play) indicates positive emotions in pigs [33–35, 46, 47].

In this study, whether the provision of toys to solicit play behaviour in pigs was associated with a change in the human-animal relationship was investigated in an experimental setting with the use of a preliminary test called the ‘strange person’ test with minipigs, a model of domestic industrial pigs [11, 36]. Our hypothesis was that the pigs could be in a more positive affective state after playing and then the valence of the human-animal relationship could be improved.

## Material and methods

The housing, husbandry and care for the animals involved in this experiment were carried out according to French and European legislation and the principles of replacement, reduction and refinement. The project, including this experimental procedure, was approved by the IRSEA’s (Research Institute in Semiochemistry and Applied Ethology) Ethics Committee (C2EA125) and the French Ministry of Research (AFCE_201609_01).

### Animals and housing

The mini-pigs (*Sus scrofa domesticus*) (*n* = 16: castrated males = 8; females = 8) involved in the study were part of a new strain resulting from the cross-breeding of miniature breeds (Asian potbelly breeds: Vietnamese and Chinese) with conventional white-hair breeds (Landrace and Large White), and they were born at the Specipig (centre for breeding and biomedical research) in Barcelona, Spain. The pigs were enrolled in this study at the age of one year. The pigs were socialised to humans since the beginning of their lives: the animal-keepers fed them every day and performed the regular cares; the veterinarians and researchers visited them regularly. They were housed in an experimental and controlled system in two identical rooms (30 m^2^) with monitored environmental parameters: a mean ambient temperature of 22 °C, the same ventilation provided by 2 artificial ventilators in each room and 60% humidity. Two pigs of the same sex and age were housed in each pen, which had the following features: an area of 2.5 m^2^ (1.85 m × 1.35 m), walls and doors with a height of 0.90 m, slatted floors, a feeder and a drinker. The mini-pigs were separated into pairs after weaning to prevent fighting, so the pigs were used to being together. The pens were cleaned daily. The lights were on from 8.00 a.m. until 6.00 p.m. The pigs were fed twice a day with a special diet for mini-pigs maintained in restricted conditions for long-term trials (Special Diets Services, Paris, France) and had continuous access to drinking water.

### Procedure

The study took place over four consecutive days; the animals were divided into two identical rooms with eight pigs in each room, and each day, only the animals in one room participated in the study. Thus, each animal participated in the study for a total of two days in two different situations: one Control session and one Play session. The tests were performed always at the same time, from 9:30 a.m. to 12:00 a.m. The pigs were not moved to other rooms or pens to participate in the study; they stayed in their own rooms and pens with their mates (in pairs) to avoid any stress that may occur from changing locations. During the Play sessions, the pigs stayed in their own pens (2.5 m^2^ of slatted floors), where two medium-sized dog toys (balls with ropes attached, Denta Fun, 7 cm/50 cm, 132 g, MonAnimalerie.net, Saint-Alexandre, France) were introduced. This type of toy was previously tested with these mini-pigs, and the toy yielded a high motivation to play when it was provided for a 10 min’ period [33]. One operator put the toys on the floor in the centre of the pen and then exited the pen, while the pigs were present in the same pen. In each pen, two toys were provided to reduce conflict due to competition. Video recordings were used to confirm that the pigs played during the Play sessions, according to previous literature [33, 34], measuring object and social play duration (Table 1). During the Control sessions, the animals were again placed in their own pens, and no toy was provided to them; this setting represented their typical housing situation, which did not have any additional stimuli. After the Control or Play session, the strange person test was performed immediately. During the test, a person wearing a colourful overall, a hood, a mask, gloves and boots (unknown person with an odd appearance) entered the pen, where 2 mini-pigs were housed, for a 2-min video recording. The person’s eyes were also covered with a dark film to prevent the pigs from seeing them. In each test, the person was dressed differently, with different colours and masks, to create novelty [26, 53]. The person was completely covered with an overall, a hood, a mask (covering also the eyes), gloves and boots, to prevent the pigs from smelling the skin or body. A tall screen was installed between the pens (the pigs were habituated to the screen) to prevent the neighbouring pigs from seeing the strange person when the test was being performed near them. Thus, the person could only be seen by the two pigs being tested. The strange person entered the pen and sat down with his or her back against the wall of the pen; the person always moved to the same position and did not move, touch or interact with the pigs in any possible way. The behaviours of each pig were recorded during the test: object play, social play, latency to approach the person, duration for which the pig was distant from the person, direct contact with the person, need to move the pig away and tail movement. All the behaviours were scored based on the continuous video recordings by two independent observers who had access to the description of each behaviour (Table 2) and an Excel matrix.

**Table 1:** Descriptive data of 600 s of the Play and Control sessions

Pig	Session	OPD	SPD
1	Play	600	112
2	Play	600	129
3	Play	584	85
4	Play	599	97
5	Play	598	47
6	Play	600	59
7	Play	543	238
8	Play	579	251
9	Play	600	166
10	Play	600	135
11	Play	600	123
12	Play	589	136
13	Play	371	59
14	Play	600	51
15	Play	600	369
16	Play	244	431

*OPD* Object play duration (in seconds), *SPD* Social play duration (in seconds)

There was no object play in the Control sessions, as no object (toy) was provided

There was no social play during the Control sessions. Both parameters (OPD and SPD) were 0 in Control sessions

**Table 2:** Description of the behaviours observed in the video analysis of the strange person test (120 s)

Behaviour	Definition
Latency to approach the person (in sec)	Duration (in seconds) that the pig takes to approach the person, without necessarily touching the person. More precisely, the number of seconds that the pig spends from the beginning of the test to cross the line that divides the pen in the “area with the person” and the “area without the person”. The perpendicular line divided the pen in two parts (1.85 m/2 = 0.93 m): the part where the strange person sat down was named the “area with the person”, and the other part was named the “area without the person”. Longitudinally, the person sat on the floor and occupied 0.55 m, meaning that when the pig crossed the line with at least one leg, the person was 0.35 m from the pig (from the pig’s leg) and thus 0.10 m from the pig’s nose.
Duration for which the pig was distant from the person (in sec)	Duration (in seconds) that the pig spends in the “area without the person” (see explanation above).
Direct contact with the person (in sec)	Duration (in seconds) that the pig touches any part of the strange person’s body with the snout.
Need to move the pig away (frequency)	Frequency (number of times) that the strange person needs to move the pig away with his or her arm or leg, nicely but firmly, because the pig is biting the person hard.
Tail movement (in sec)	Duration (in seconds) of tail swinging in any direction; the tail mostly was swinging from side to side, resulting in lateral tail movements [31, 34, 46].

Behaviours could occur simultaneously and were not mutually exclusive

### Statistical analysis

Data analysis was carried out using SAS 9.4 software (Copyright (c) 2002–2012 by SAS Institute Inc., Cary, NC, USA). The significance threshold was fixed at 5%, which is a standard threshold.

The reliability between the two observers who assessed the video recordings was calculated using the CORR procedure and Spearman’s or Pearson’s correlation coefficients, depending on the normality of data (normality was verified using the UNIVARIATE procedure): Spearman’s correlation coefficient was used when normality was not verified for at least one variable, and Pearson’s correlation coefficient, when normality was verified for both variables. This methodology was selected according to a study by Martin and Bateson [38].

Comparisons of the strange person test results after the Play and Control sessions were performed using mixed models, and the pen was considered a random effect. The following parameters were studied: latency to approach the person (duration in seconds), duration for which the pig was distant from the person (duration in seconds), duration of direct contact (duration in seconds), the need to separate the pig (in frequency) and duration of tail movement (duration in seconds).

For the continuous variables (latency to approach the person, duration for which the pig was distant from the person, duration of direct contact and duration of tail movement), the distribution of the variable and the model residuals were analysed using the UNIVARIATE procedure to determine whether the assumption of normality was met. If normality was verified, the raw data were used for the model using a general linear mixed model with the MIXED procedure. If the data for the variable did not follow a normal distribution, other continuous distributions were tested with the UNIVARIATE procedure. When a distribution was identified, the data were modelled with the corresponding generalised linear mixed model using the GLIMMIX procedure. When no distribution was identified, a Box-Cox transformation was applied with the TRANSREG procedure to reach normality. Whether a general linear mixed model or generalised linear mixed model was used, the best structure of the covariance matrix was selected by minimising the AICC and BIC criteria.

For the discrete variable (the need to separate the pig, in frequency), the distribution of the data was analysed to identify the best discrete distribution for modelling the data. Because the data distribution corresponded to a Poisson distribution, a Poisson mixed model was generated using the GLIMMIX procedure and the LAPLACE method for estimating parameters. When the Poisson model showed overdispersion, a negative binomial mixed model was preferred.

## Results

### Inter-observer reliability

The reliability between the two observers who performed the video analysis was calculated using Spearman’s or Pearson correlation coefficients (Table 3). The reliability (inter-observer agreement) was high for all the parameters.

**Table 3:** Inter-observer reliability between the two observers carrying out the video analysis

Parameter	Correlation Coefficient	***P***-value
Object Play Duration	Spearman: rho = 0.90	< 0.0001
Social Play Duration	Spearman: rho = 0.97	< 0.0001
Latency to approach the person	Spearman: rho = 0.95	< 0.0001
Duration for which the pig was distant from the person	Spearman: rho = 0.93	< 0.0001
Direct contact	Spearman: rho = 0.93	< 0.0001
Need to separate the pig	Spearman: rho = 0.98	< 0.0001
Tail movement	Pearson: r = 0.90	< 0.0001

### Comparisons between the strange person test results after the play and control sessions

Comparisons between the strange person test results after the Play and Control sessions were analysed for all parameters. The video recordings confirmed that the pigs performed play behaviour during all Play sessions and that they did not perform play behaviour (neither object play nor social play) during Control sessions (Table 1).

#### Latency to approach the person (duration in seconds)

A significant difference was found between the strange person test results after the Play and Control sessions (Degrees of Freedom = 30; Statistic of the F test = 39.1; *p* < 0.0001; generalised linear mixed model for a lognormal distribution). More precisely, the latency to approach the person was significantly lower after the Play sessions than after the Control sessions (Table 4).

**Table 4:** Parameters (behaviours) analysed during the strange person test (total of 120 s): comparison between the test results after the Play sessions and after the Control sessions

Parameter	Session	N	Mean	Std Dev	Median	Minimum	Maximum	***P***-value
Latency to approach the person (sec)	Play	16	4.9	5.0	3.5	1.5	19.0	< 0.0001*
	Control	16	40.9	43.1	26.5	2.0	120.0	
Duration for which the pig was distant from the person (sec)	Play	16	4.5	6.4	1.3	0.0	23.5	< 0.0001*
	Control	16	43.4	43.5	31.8	1.5	120.0	
Direct contact duration (sec)	Play	16	85.8	24.9	91.75	17.0	116.0	0.002*
	Control	16	55.8	38.0	64.0	0.00	111.0	
Need to separate the pig (frequency)	Play	16	6.1	5.2	3.8	1.0	16.0	0.005*
	Control	16	1.6	1.7	1.5	0.0	6.0	
Tail movement duration (sec)	Play	16	92.8	26.8	104.5	28.5	119.5	0.003*
	Control	16	60.0	34.1	61.0	0.0	114.0	

*Significant differences

#### Duration for which the pig was distant from the person (duration in seconds)

A significant difference was observed between the strange person test results after the Play and Control sessions (Degrees of Freedom = 15; Statistic of the F test =54.3; *p* < 0.0001; general linear mixed model). The duration for which the pig was distant from the person was significantly lower after the Play sessions than after the Control sessions (Table 4).

#### Direct contact (duration in seconds)

A significant difference was found between the strange person test results after the Play and Control sessions (Degrees of Freedom = 15; Statistic of the F test = 14.8; *p* = 0.002; general linear mixed model). The duration of direct contact was significantly higher after the Play sessions than after the Control sessions (Table 4).

#### Need to move the pig away (frequency)

A significant difference was found between the strange person test results after the Play and Control sessions (Degrees of Freedom = 30; Statistic of the F test = 9.3; *p* = 0.005; generalised linear mixed model for a Poisson distribution). The need to move the pig away was significantly higher after the Play sessions than after the Control sessions (Table 4).

#### Tail movement (duration in seconds)

A significant difference was found between the strange person test results after the Play and Control sessions (Degrees of Freedom = 15; Statistic of the F test = 12.6; *p* = 0.003; general linear mixed model). The tail movement was significantly more frequent after the Play sessions than after the Control sessions (Table 4).

## Discussion

This exploratory study aimed to investigate whether the provision of toys adapted to pigs to allow them to perform play behaviour was associated with a change in the human-animal relationship and their emotional state. This association was tested with a preliminary and simple test named the ‘strange person’ test, which was adapted to the present conditions.

Overall, the results suggest an improvement in the human-animal relationship after Play sessions: the pigs seemed to be less fearful, more curious and more inclined to interact with the strange person, showing a more positive emotional state based on an increase in tail movement duration (e.g., [34]). This result suggests that the provision of toys, and more precisely, the opportunity to perform object play behaviour, and sometimes, spontaneously, social play behaviour, can improve the human-animal relationship and the positive emotions of pigs.

The latency to approach the person and the duration for which the pig was distant from the person reflected the fearfulness of the animals [27] towards the strange person as well as their emotional state [3] because of two factors: the person was unfamiliar and the situation was strange for the pigs, as the person was dressed up and did not interact at all with them, which were both strange aspects for the pigs. The results showed that the latency to approach the strange person and the time the pigs spent distant from the person were shorter after playing than after the Control situation, suggesting that the animals were less fearful and in a more positive emotional state, as they were more inclined to approach the person. The results may even suggest that the animals were in a more optimistic state [41], as it can be interpreted that they preferred to approach something new rather than remain in a safe place far from it. Some studies have shown that the provision of environmental enrichment can improve the human-animal relationship and thus their approach towards humans and the ease of working with and handling them (e.g., [49]). Nevertheless, other studies suggested that enrichment objects do not affect the ease of handling pigs [7, 29]. Thus, van de Weerd and Day [56] suggested that the type of enrichment and quantity of stimuli provided influences the extent of the effect on their behaviour towards humans. This preliminary study provides more information about this topic.

The duration of direct contact with the person, which is also related to the absence or presence of fearfulness, is also linked to exploratory behaviour, as we defined it as the duration of time that the pig spent touching the strange person with the snout. By watching the video recordings thoroughly (data not shown), we observed that the most common behaviour performed by the pigs during direct contact with the person was explorative behaviour, or more precisely, the following behavioural elements: rooting, chewing and sniffing [52]. When pigs explore their surroundings, they do it with different purposes, such as to find food or a place to lie (appetitive behaviour, also named extrinsic exploration) or to obtain general information on their surroundings (intrinsic exploration) [2, 62], as in the case of the present study. Intrinsic exploration is motivated by curiosity, and it serves to keep the pig informed about the environment and the resources available in it [52]. Curiosity has been considered a positive feeling for the animals by several authors [17, 40–42], and according to the affective state orientation, an animal’s welfare is positive when it adapts with positive emotional experiences and/or without negative experiences during its interactions with other animals, people and the environment [14]. Thus, this result suggests that pigs were more inclined to interact with the person after playing (improvement of the human-animal relationship) and that they seemed to be in a more positive emotional state.

The need to move the pig away was significantly more frequent after the Play sessions than after the Control sessions. This parameter may be linked to the confidence of the pig and thus to their emotional state, as well as to the motivation to explore and to interact with the person. The need to move the pig was more frequent after playing, and this result supports previous findings; thus, the pigs seemed to be in a more positive emotional state and more motivated to interact with the person, even when the person was strange and unfamiliar. Nevertheless, in this case, it was a ‘negative’ aspect for the human, as it suggests that the pig was too confident and then it may be dangerous when interacting with humans, as suggested with other species like horses [9, 13]. Of course, it is a preliminary test, the results of which should be interpreted with caution, but the results seem to be in agreement with those of other studies in different species.

Finally, regarding tail movement duration, we found that the duration was significantly longer after play than after the control conditions. Some studies showed a link between an increase in tail movement duration in pigs and play behaviour or the interaction with some types of enrichment material [33, 34, 37]. In the present study, this behaviour was significantly higher after playing, not during it, which is a new finding. It has also been suggested to be an indicator of positive emotions (or an indicator of emotions with a positive valence or outcome) by several studies conducted in different conditions [33–35, 37, 46, 47]. The present significant difference supports the previous results about the other parameters, suggesting that the pigs were in a more positive state after playing during the strange person test than after a control situation based on the increase in tail movement duration.

Many authors have highlighted the need for more research to identify indicators of emotional states [3, 10, 39], particularly of emotional valence, to better assess animal welfare [15]. The present study also provides more information about some indicators of positive emotions, which may be useful to improve animal welfare assessments in a feasible way, as well as some preliminary tools to assess the human-animal relationship.

Some limitations of the study would be the sample size and the use of mini-pigs instead of domestic commercial pigs. Nevertheless, these controlled and experimental conditions allowed us to obtain significant results, which open some doors for future studies.

Finally, this preliminary study suggests that the provision of toys to pigs can improve the human-animal relationship, which, in combination with other methods, such as being nice to the pigs, may be a feasible and simple method of improving this relationship. Additional research to explore this concept thoroughly may yield interesting results because a positive emotional state of the animals and a good human-animal relationship are essential to ensure the quality of life of farm animals as well as the quality of the final product. Other factors may be of interest to study, like the potential sex and/or hormonal differences in some behaviours, like in fear reactions, as already shown in other species like sheep [1, 55]. Future studies with domestic commercial pigs in farm conditions may be of interest, with an adaptation of the ‘strange person’ test, in order to investigate if the results would be the same than in the experimental setting with mini-pigs.

## Conclusions

In conclusion, this exploratory study suggests that play behaviour due to the provision of toys seems to improve the human-animal relationship and the emotional state of pigs. More research in farm conditions, with this method and other methods, should be performed to provide more information about this potentially interesting and feasible method of improving the human-animal relationship and emotional state of pigs.

## Data Availability

The datasets during and/or analysed during the current study available from the corresponding author on reasonable request.
